# Use of eye tracking in analyzing distribution of visual attention among critical care nurses in daily professional life: an observational study

**DOI:** 10.1007/s10877-020-00628-2

**Published:** 2020-12-09

**Authors:** Daniel A. Hofmaenner, Anique Herling, Stephanie Klinzing, Stephan Wegner, Quentin Lohmeyer, Reto A. Schuepbach, Philipp K. Buehler

**Affiliations:** 1 Institute of Intensive Care Medicine, University Hospital Zurich, Raemistr. 100, 8091 Zurich, Switzerland; 2 Department of Mechanical and Process Engineering, ETH Zurich, Zurich, Switzerland

**Keywords:** Eye tracking, Visual attention, Patient safety, Human errors, Work patterns

## Abstract

Patient safety is a priority in healthcare, yet it is unclear how sources of errors should best be analyzed. Eye tracking is a tool used to monitor gaze patterns in medicine. The aim of this study was to analyze the distribution of visual attention among critical care nurses performing non-simulated, routine patient care on invasively ventilated patients in an ICU. ICU nurses were tracked bedside in daily practice. Eight specific areas of interest were pre-defined (respirator, drug preparation, medication, patient data management system, patient, monitor, communication and equipment/perfusors). Main independent variable and primary outcome was dwell time, secondary outcomes were hit ratio, revisits, fixation count and average fixation time on areas of interest in a targeted tracking-time of 60 min. 28 ICU nurses were analyzed and the average tracking time was 65.5 min. Dwell time was significantly higher for the respirator (12.7% of total dwell time), patient data management system (23.7% of total dwell time) and patient (33.4% of total dwell time) compared to the other areas of interest. A similar distribution was observed for fixation count (respirator 13.3%, patient data management system 25.8% and patient 31.3%). Average fixation time and revisits of the respirator were markedly elevated. Apart from the respirator, average fixation time was highest for the patient data management system, communication and equipment/perfusors. Eye tracking is helpful to analyze the distribution of visual attention of critical care nurses. It demonstrates that the respirator, the patient data management system and the patient form cornerstones in the treatment of critically ill patients. This offers insights into complex work patterns in critical care and the possibility of improving work flows, avoiding human error and maximizing patient safety.

**Supplementary Information:**

The online version of this article (10.1007/s10877-020-00628-2) contains supplementary material, which is available to authorized users.

## Introduction

The field of intensive care medicine is complex and challenging. Healthcare professionals working in intensive care units (ICU) are expected to work under high physical and mental stress involving multi-tasking and interdisciplinary knowledge [1]. Furthermore, an increasing number of monitoring and technical assistance devices are available on the market and influence operational procedures, situational awareness (“the perception of elements of the environment within a volume of time and space” [2]), communication and interaction with coworkers and critically ill patients [3, 4].

In line with these developments, patient safety has emerged as a priority in intensive care medicine [5]. Due to the severity and complexity of their disease, especially invasively ventilated critical care patients seem to be more vulnerable to critical incidents such as medication errors, medical interventions or other iatrogenic harm [6, 7]. Therefore, all work processes carried out in the ICU should be structured, carefully planned and frequently reassessed.

To date, it is unclear how sources of human error and complex work patterns in the ICU should best be analyzed to minimize harm to patients, improve patient safety and make work processes more efficient. It is likewise unclear what influence monitoring and technical assistance machines can have on work processes. One way to analyze these are bedside observations of real-life workflows [7–9]. However, such observations have limitations, the greatest of which is possible observation bias. This makes the use of direct observation in the setting of intensive care medicine less than ideal and there is a lack of knowledge about the particular importance of visual behavior related to clinical tasks, especially with regards to the complexity of invasively ventilated ICU patients.

On the other hand, eye tracking has been found useful in analyzing gaze patterns and visual attention in different fields of medicine [10–17], as it enables investigating how healthcare professionals react to different verbal or non-verbal messages and helps to understand their cognitive engagement in real time. Eye tracking involves the analysis of eye movements and the behaviors of the pupils by using infrared lights reflected by the cornea and detected by cameras. Gaze metrics provide indices to assess visual patterns of participants. It minimizes recall errors and effects related to expected behavior, while revealing information conventional observation research methods normally miss.

The aim of this study was therefore to analyze the distribution of visual attention among critical care nurses performing non-simulated, routine patient care on invasively ventilated patients in an ICU.

## Methods

### Study design

This was a prospective, observational single-center study conducted at the Intensive Care Unit (ICU) of the University Hospital of Zurich (Zurich, Switzerland) between September 2018 and April 2019. At this interdisciplinary ICU, which houses 64 beds, around 4000–4500 patients are admitted and treated per year. The relevant local ethics committee (Kantonale Ethikkommission Zurich BASEC ID REQ 2017-00798) approved the study in accordance with the Helsinki Declaration. Provided informed consent was given and no exclusion criteria were fulfilled, all nurses working in the ICU were eligible for the study regardless of their professional experience. Exclusion criteria were: declared impaired vision (lack of stereoscopic vision, monocular vision or achromatopsia), withheld informed consent or imprecise recordings that could not be analyzed by the software or computer (e.g. lack of accuracy of the visual fixation, impossibility of calibration, blurred images, software not being able to assign visual fixation). If criteria of imprecise recordings were fulfilled, the participant was excluded.

We provided special correction glasses (fabricated by SensoMotoric Instruments, Teltow, Germany), if necessary.

### Recruitment

Participation in the study was voluntary and free of charge.

The study was designed to include at least 25 ICU nurses. In view of possible dropouts, 30 participants were recruited in total. They were either certified ICU nurses or ICU trainees during their vocational education. After signing the informed consent, all participants were given a written and oral introduction to the task and aim of the study. The participants were included in the study if technical calibration of the eye tracker was accurate and no other exclusion criteria were met. Because all patients involved were intubated, the patients' legal representatives had to give written informed consent as well.

### Data recording

An SMI eye tracking glasses 2 wireless system (SensoMotoric Instruments, Teltow, Germany) was used for the recordings in the present study. This device has a sampling rate of 60 Hz and measures angles of view for all distances with an accuracy of 0.5°. Resolution of the recorded scene video is 960 × 720 px at 30 fps. Raw data were analysed using the SMI BeGaze 3.6 software (SensoMotoric Instruments, Teltow, Germany) with its integrated algorithm for fixation determination.

### Task

Prior to the eye tracking measurements, participants were asked to fill-in a pre-experiment questionnaire including demographic data, professional experience and visual impairments. Subjective health status (using a numerical scale 0–10) and current workload (using a numerical scale 0–20) were assessed, as they might influence eye tracking data.

After habituation to the eye tracker, a three-point calibration was performed. After calibration, each participant was recorded with the eye tracker in his/her daily, professional life on the ICU while caring for his/her patient(s). Targeted recording time was 60 min per participant. The invasively ventilated critical care patients were from different medical fields, including internal medicine, neurology, traumatology, thoracic and visceral surgery, transplantation medicine, gynecology, urology and plastic surgery. No patient was intubated only for the study purposes, the reason for being ventilated was the medical condition. All patients were ventilated by Hamilton S1 respirators (Hamilton Company, Reno, Nevada, USA). All patients had the same intravascular accesses.

All participants were instructed to behave as they would under normal circumstances and to perform all necessary professional activities as if they were not being tracked. No special task was given to the participants. All recordings occurred in the early afternoon, to avoid biases by the doctor’s visit in the morning. No recordings were performed during nighttime to avoid confounding.

In this way, direct patient care, communication (with colleagues/patients/relatives) and technical handling (e.g. handling of ventilator, preparing a syringe, adjusting perfusors) could be recorded and analyzed with respect to their temporal distribution.

After the recordings, a post-experiment questionnaire was filled-in.

### Data analysis

The distribution of nurses’ visual attention in their everyday professional life was analyzed. In total, eight specific areas of interest (AOI, areas being important to provide a comprehensive picture of everyday situations in the ICU) were defined for analysis. The relevant AOIs were defined prior to the recordings, were chosen according to the assumed clinical relevance of them and were: respirator, drug preparation, medication (e.g. preparing and application of intravenous drugs), patient data management system (PDMS, MetaVision iMD*soft*, Israel, used for documentation and computerized physician order entry), patient, monitor (vital signs, including vital signs obtained by PDMS), communication (to other healthcare professionals and family members) and equipment/perfusors. The remaining fixations were classified as “not relevant” (e.g. white space, gaze patterns for spatial orientation, floors, roofs and AOIs other than the previously defined).

### Primary outcome

Main independent variable for these AOIs and primary outcome was dwell time (cumulated time spent on an AOI, including fixations, blinks and saccades, a marker of the importance of the AOI).

### Secondary outcomes

Secondary outcomes were hit ratio (percentage of participants gazing at a particular AOI), revisits (cumulated number of revisits to a particular AOI, a marker of complex or significant visual perception), fixation count (cumulated number of gaze fixations on a particular AOI) and average fixation time on an AOI (a marker of the complexity of an AOI).

### Statistics

Results are expressed as percentages for categorical variables and as median and interquartile range (25–75th percentile) for continuous variables. A p-value was considered statistically significant when < 0.05.

Chi-square or Fisher exact tests were used for discrete variables. Multiple comparisons were performed using the Friedman’s test, with Dunn’s correction.

Statistical analysis was performed using SPSS Version 23 (SPSS Science, Chicago, IL, USA), Graphpad prism 7 (San Diego, CA, USA), and Microsoft Excel (Microsoft Office Professional Plus 2013; Microsoft Corporation, Redmond, WA, USA).

## Results

30 participants were recruited for the study. Due to technical deficiencies in the recordings, two participants had to be excluded from the analyses. Data gathering for the 28 included participants occurred without any technical problems. Total tracking time was 1837 min (on average 65.6 min per participant). 85.7% of participants were female, the median of professional experience as nurse was 18 years. The median of ICU experience was 11.5 years. Baseline characteristics of participants are presented in Table 1.

**Table 1:** Baseline characteristics of participants

Baseline characteristics	
Age	
Years	39.5 (29–45.5)
Sex	
Male	4 (14.3%)
Female	24 (85.7%)
Vision correction	
No	17 (60.7%)
Yes	11 (39.3%)
Professional experience total	
Years	18 (5.5–25)
Professional experience ICU	
Years	11.5 (3–16.5)
Being rested^a^	
(Scale 0–10)	7 (6–8)
Subjective health^a^	
(Scale 0–10)	9 (8–9)
Mental workload before tracking^a^	
(Scale 0–20)	12.5 (10–14.8)
Physical workload before tracking^a^	
(Scale 0–20)	10.5 (8–12.5)
Mental workload during tracking^a^	
(Scale 0–20)	12.5 (6.3–14)
Physical workload during tracking^a^	
(Scale 0–20)	7.3 (5.5–11)
Subjective stress during tracking^a^	
(Scale 0–10)	4 (2–5)

Data expressed as number (%) or median and interquartile range (IQR); subjective health status (using a numeric scale where 0 = totally sick and 10 = normal health) and current workload (using a numeric scale where 0 = totally relaxed and 20 = totally stressed)

^a^Marks subjective/self-assessed characteristics

In the pre-experiment questionnaire, the median self-assessed mental and physical workload prior to the experiment was 12.5 and 10.5 respectively, and 12.5 and 7.3 during the experiment in the post-experiment questionnaire (on a scale of 0–20, with 0 indicating no workload). In the recordings, all pre-defined AOIs were hit by all participants (i.e. a hit ratio of 100% for all AOIs).

**Fig. 1:**
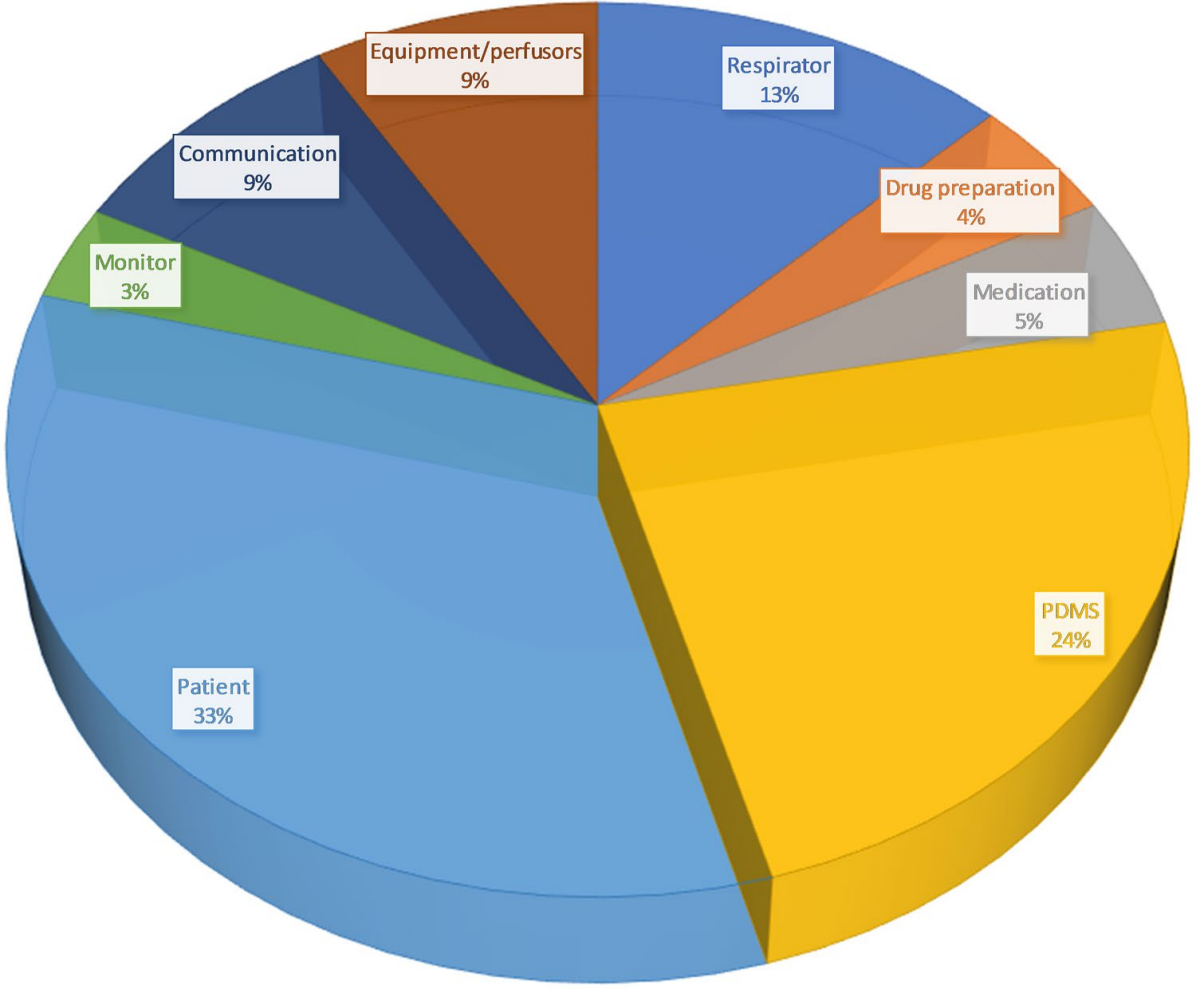
Dwell time on each predefined AOI in percent. Fixations on the AOI “not relevant” are not included

**Fig. 2:**
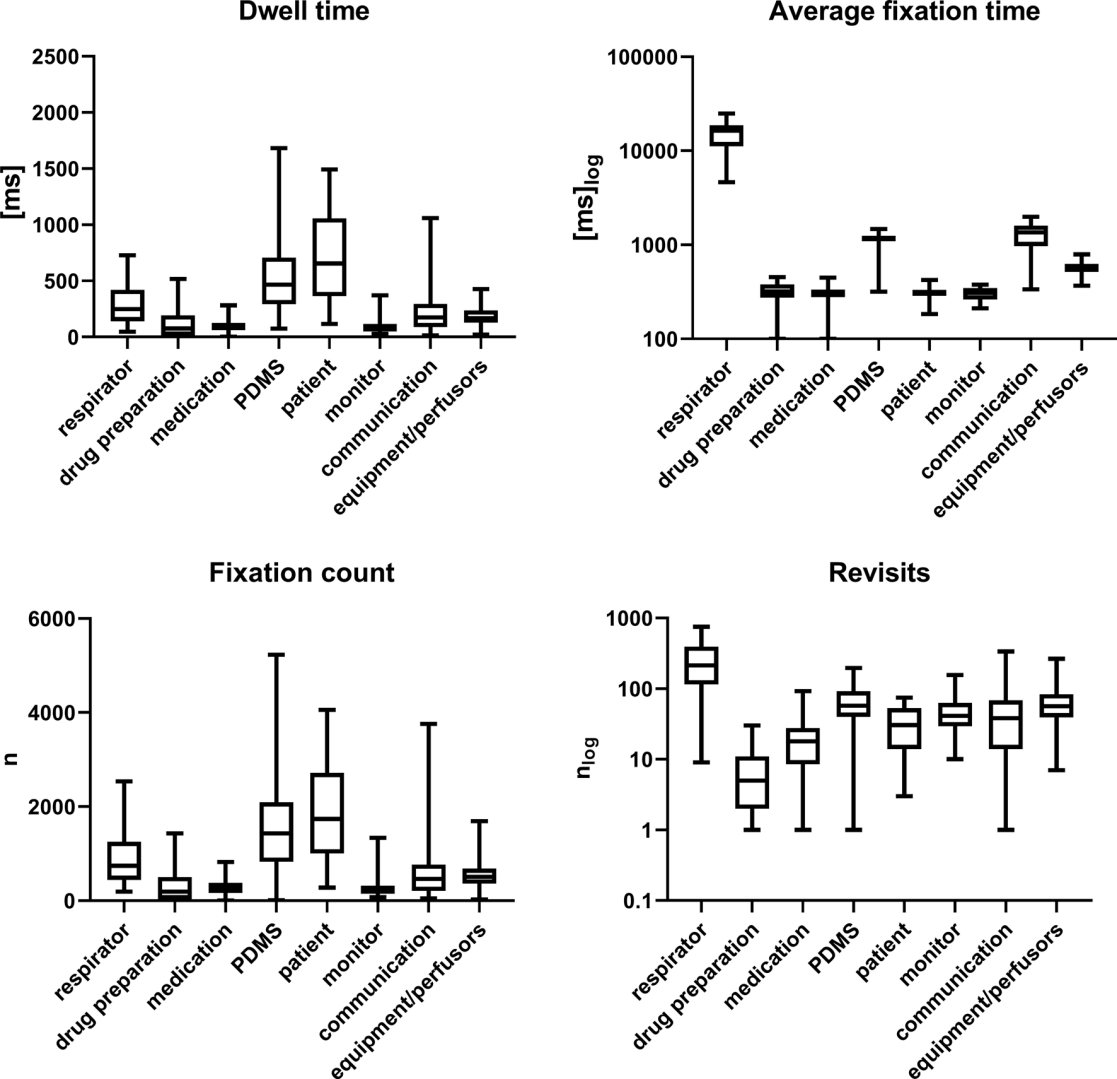
Box plots indicating Dwell time, fixation count, average fixation time and revisits for AOIs. P-values for multiple comparisons are provided in the Supplementary Table 1

Figure 1 shows the primary outcome dwell time on each predefined AOI in percent. Table 2 and Fig. 2 provide an overview of dwell time, fixation count, average fixation time and revisits to the different AOIs. Compared to the other AOIs, dwell time was significantly higher (p < 0.05) for the AOIs respirator (12.7% of total dwell time), patient data management system (23.7% of total dwell time) and patient (33.4% of total dwell time). A similarly significant distribution was observed for fixation count (respirator 13.3%, patient data management system 25.8% and patient 31.3%) (Table 2 and Fig. 2).

**Table 2:** Dwell time, average fixation time, fixation count and revisits for different areas of interest

	Dwell time [s]	Average fixation [ms]	Fixation count [n]	Revisits [n]
Respirator	248 (140.8–412.2) [156.8–391.1]	16,337.3 (11,597.5–18,598.1) [14031.1–17,361.9]	740 (453.5–1237.5) [501–1169]	214 (117–392) [141–372]
Drug preparation	88.3 (49.7–200.4) [57.3–172.3]	319.5 (294.4–382.1) [303.8–366.8]	203 (110–526) [149–438]	6 (2–11) [3, 11]
Medication	93.3 (67.2–126.3) [75.9–121.6]	300.7 (278.4–333.9) [281.7–318.7]	255 (175–381) [216–339]	19 (10–28) [16–26]
PDMS	464.8 (293.9–706.7) [341.5–670.6]	1151.2 (1095.5–1239.9) [1117.6–1191.5]	1431 (826–2077.5) [967–1898]	57 (40.5–90.5) [48–86]
Patient	654.6 (375.3–1038) [502.5–941.1]	315.2 (286.1–330.2) [295–324]	1739 (1004–2669.5) [1284–2361]	30.5 (14–51) [18–45]
Monitor	67.6 (50.1–115.6) [61–110.4]	308.3 (263.7–346.2) [279.2–343.7]	220 (145.5–314) [170–297]	41 (29.5–61) [32–53]
Communication	173.2 (89.3–291.8) [107.6–240.5]	1356.6 (974.3–1597.2) [1138.9–1555.2]	462 (213.5–754) [232–529]	38 (14–67.5) [20–54]
Equipment/perfusors	168.6 (127.8–230.9) [150–201.9]	578.3 (515.4–623.7) [532.3–601.9]	506 (365.5–680) [396–595]	56.5 (39.5–83) [43–69]
Not relevant	634.6 (475.5–864.1) [532.3–796.5]	470.4 (290–540.6) [360.1–508.7]	1996 (1411.5–2523) [1536–2123]	102.5 (70.5–142.5) [84–127]

Data expressed as median, (Interquartile Range) and [95% confidence interval (CI)]

**Fig. 3:**
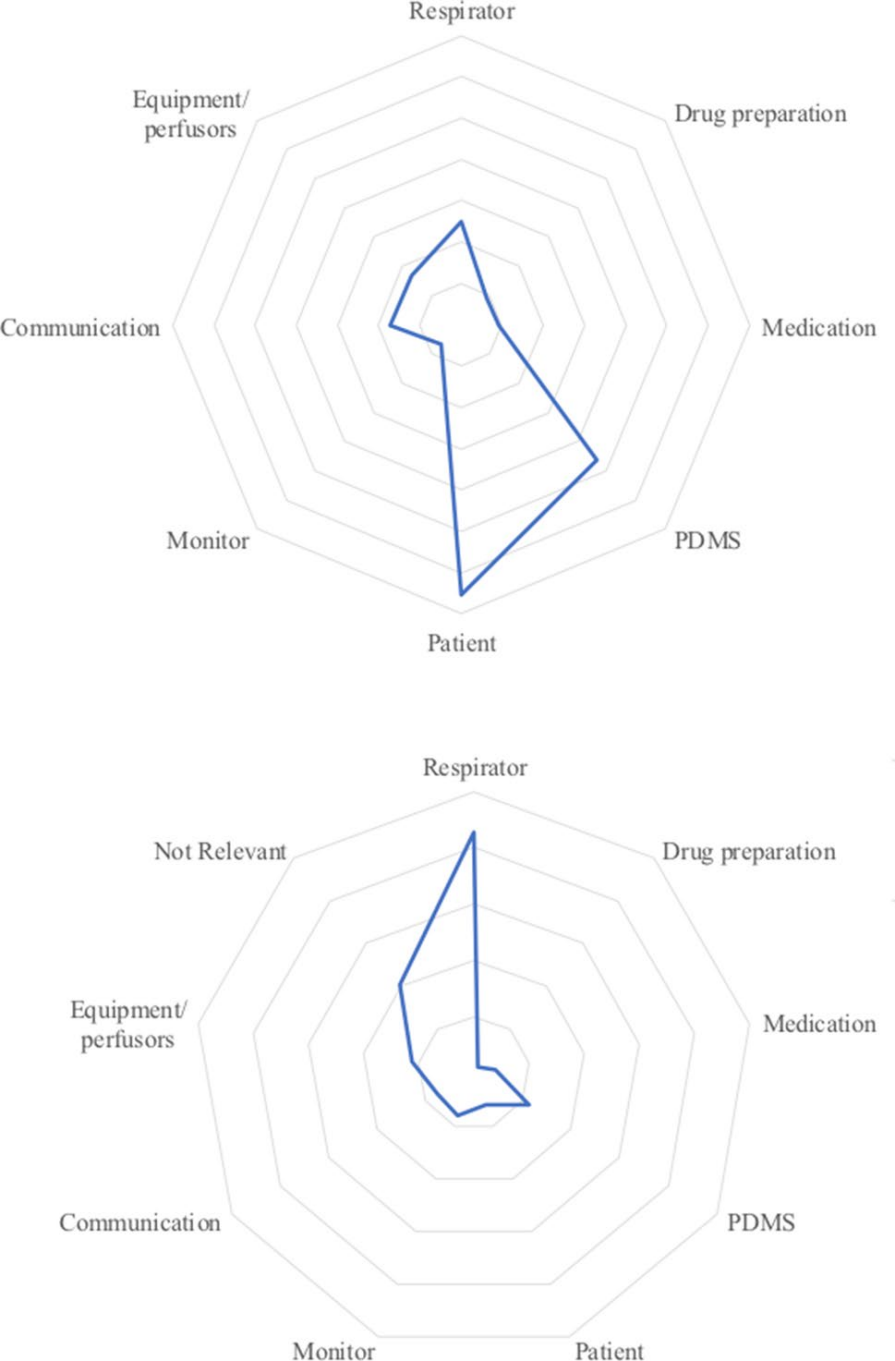
Spider diagram indicating Dwell time (upper **a**) and Revisits (lower **b**). Upper figure: one line represents 100 s; lower figure: one line represents 50 Revisits

Across all AOIs, average fixation time and revisits to the respirator were markedly elevated. Apart from the respirator, average fixation time was highest (statistically significant, p < 0.05) for PDMS, communication and equipment/perfusors. Figure 3a shows the two significantly increased dwell times (patient, PDMS) in a spider diagram. The distribution of revisits is shown in Fig. 3b.

Supplementary Table 1 provides all p-values for multiple comparisons.

## Discussion

The aim of this study was to analyze the distribution of visual attention among critical care nurses performing non-simulated, routine patient care on invasively ventilated patients in an ICU.

Eye tracking proved to be easily feasible and safe for patients and employees. No critical incidents or patient harm occurred during the measurements and no recordings had to be interrupted by the study team owing to safety concerns. Overall, this study showed that eye tracking is a helpful tool in measuring and quantifying the distribution of visual attention among critical care nurses in an objective way and in assessing the complexity or the importance of professional work tasks. Owing to the long tracking time and adequate number of participants, a realistic picture of daily situations in the ICU could be obtained. A biasing influence due to differences in nurses’ workloads could be excluded, because the subjective workloads reported in the questionnaires were similar. Certain factors such as documentation (24%) or patient care (33%) proved to be relatively constant.

The main results suggest that the pre-defined AOIs carry different importance in patient care on the ICU (Table 2, Figs. 1 and 2). Specifically, Patient Data Management Systems (PDMS), patient care and the ventilator attracted the most visual attention from nurses.

Compared to other AOIs, PDMS showed a significantly increased dwell time. This underlines the importance of mandatory tasks related to documentation, which might shift nurses’ activity from nursing to administration. Due to a probable increased workload in the area of documentation, nursing and monitoring activities might be reduced to an extent that could have an impact on patient safety. Furthermore, high revisit rates and average fixation times show not only that the quantitative duration of time spent on PDMS has possibly increased, but also that information acquisition and processing is likely becoming more difficult. Long fixation times and high revisit rates indicate complex information absorption and can lead to staff fatigue/alarm fatigue with consecutive loss of attention and subsequent errors. Therefore, despite advantages, such as reduced paperwork or electronically stored data, uncritical use of PDMS might expose patients to further risks (e.g. neglecting patient care, risk of missing alarms while glancing at computer monitors). In our opinion, the operator complexity of electronic data systems should probably be reduced and the handling simplified.

Contrary to our expectations, we were able to demonstrate that visual monitoring of vital signs (i.e. the AOI monitor) accounted for only 3% of the cumulative dwell time and that it was not frequently fixed in the other analyses. Similar findings were made by Law et al. in another non-simulated eye tracking study [17], where the authors argued that frequent looks at monitors displaying unchanging information, especially when auditory alarms are available, might be inefficient. These findings underlie the importance of narrow alarm limits on the monitoring systems and the importance of acoustic alarms. However, poorly or incorrectly set alarm systems can also lead to alarm fatigue and affect patient safety.

Significantly, despite all the devices, the patient is still accorded great visual significance in modern intensive care medicine. Dwell time and fixation counts for this AOI were elevated and mirror the well-known intensity in nurse-to-patient contacts. One reason could be the large amount of non-verbal information, which still accounts for a large part of the communication, care and interaction between nurses and patient despite the frequent use of machines.

Finally, dwell time, fixation count, average fixation time and revisits were higher for the AOI respirator, compared to the other AOIs. Since all patients were mechanically ventilated, this is a plausible result. However, this also shows that using and handling a respirator can pose a challenging task for professionals, in line with the fact that little is known about the visual attention of nurses while using this device. This was underlined by the impressive elevation of average fixation time and revisits to the AOI respirator, which might suggest increased operator complexity and subsequently increased risks of operating errors. In our eyes, this finding carries the risk of visual absorption and possibly neglecting other important aspects of patient care. Further studies are warranted to investigate visual attention and gaze patterns when using respirators.

One advantage of this study is that it is one of the first to examine visual attention in a real-life situation in an ICU by using eye tracking [18]. A few studies have used eye tracking in critical situations, but these were simulated [6, 7, 10–15, 19–23]. Eye tracking can also be successfully integrated into electronic health record-based simulation and provides a surrogate measure of cognitive decision-making and electronic health record usability [21]. However, Grundgeiger et al. showed that simulated data differ from real work environments and highlighted the need for caution when translating simulation-based research to topics involving visual attention to the real clinical environment [11]. Another advantage of this study is its demonstration that by uncovering and understanding socio-technical systems and human–machine interactions, patient safety might be influenced. Research in human–computer interaction in the field of critical care has the potential to improve usability of user interfaces. The data from this study can be used to design further studies with a controlled design and to investigate visual perception in real-life situations in intensive care.

This study also has several limitations. The difficulty to link gaze patterns with cognition is a major limitation of the eye tracking technology. However, currently there might be no better tool to evaluate cognitive complex procedures in real-life. Moreover, participants’ knowledge of the aim of the study might have been a bias. The recruited patients were from different fields of medicine. As a consequence, each clinical scenario and associated nursing tasks might have differed, despite the fact that all patients were intubated. In addition, no pre-defined tasks were given to the participating nurses, which might have influenced comparability of data. Furthermore, recordings were performed at different times of day and routine ward rounds were not recorded. The single-center design could also have influenced the data.

Further studies are needed to investigate the benefit of eye tracking in analyzing visual attention in Critical Care. Specifically, its role in running technical devices (e.g. respirators, PDMS) and associated effects on patient safety remain to be elucidated, as this probably has an influence on patient outcome. Additionally, eye tracking might help to gain deeper insights into workflows and communication patterns in the ICU, which could subsequently be optimized and structured. It can help to identify potentially harmful patterns such as inadequate visual fixation and distractibility during high-risk procedures, which could be addressed in future studies.

Overall, eye tracking is a useful tool for analyzing the distribution of visual attention by critical care nurses as well as human–machine interactions in realistic professional scenarios.

This study demonstrates that the main AOIs—respirator, PDMS and the patient—form the cornerstones in the complex treatment of invasively ventilated patients in the ICU. This finding potentially offers new insights into complex work patterns in critical care medicine and the chance to improve work flows, avoid human errors and maximize patient safety.

## Supplementary Information

Below is the link to the electronic supplementary material.

Supplementary Information 1 (DOCX 15 kb)

## Data Availability

All data are transparent and strictly anonymized.
