# Supplementary material for: Use of eye tracking in analyzing distribution of visual attention among critical care nurses in daily professional life: an observational study
Source: J Clin Monit Comput. 2020 Dec 9;35(6):1511–8. doi: 10.1007/s10877-020-00628-2 (PMC7724778; doi:10.1007/s10877-020-00628-2)
Supplement: Supplementary file 1 — Supplementary Information 1 (DOCX 15 kb) [file 10877_2020_628_MOESM1_ESM.docx]

| **Supplementary Table 1** | |  |  |  |  |  |  |  |
| --- | --- | --- | --- | --- | --- | --- | --- | --- |
|  |  |  |  |  |  |  |  |  |
|  | **Dwell Time** | | **Average Fixation Time** | | **Fixation count** | | **Revisits** | |
| **Dunn's multiple comparisons test** | **Adjusted P Value** | **Summary** | **Adjusted P Value** | **Summary** | **Adjusted P Value** | **Summary** | **Adjusted P Value** | **Summary** |
| **drug preparation vs. respirator** | 0.0017 | ** | <0.000001 | **** | <0.0001 | **** | <0.0001 | **** |
| **medication vs. respirator** | 0.0034 | ** | <0.000001 | **** | 0.0007 | *** | <0.0001 | **** |
| **PDMS vs. respirator** | >0.9999 | ns | 0.247205 | ns | >0.9999 | ns | 0.0575 | ns |
| **patient vs. respirator** | 0.1511 | ns | <0.000001 | **** | 0.8147 | ns | <0.0001 | **** |
| **monitor vs. respirator** | 0.0002 | *** | <0.000001 | **** | 0.0002 | *** | 0.0007 | *** |
| **communication vs. respirator** | >0.9999 | ns | >0.999999 | ns | 0.6601 | ns | <0.0001 | **** |
| **equipment vs. respirator** | >0.9999 | ns | 0.000215 | *** | >0.9999 | ns | 0.1171 | ns |
| **medication vs. drug preparation** | >0.9999 | ns | >0.999999 | ns | >0.9999 | ns | 0.6145 | ns |
| **PDMS vs. drug preparation** | <0.0001 | **** | 0.000001 | **** | <0.0001 | **** | <0.0001 | **** |
| **patient vs. drug preparation** | <0.0001 | **** | >0.999999 | ns | <0.0001 | **** | 0.0072 | ** |
| **monitor vs. drug preparation** | >0.9999 | ns | >0.999999 | ns | >0.9999 | ns | <0.0001 | **** |
| **communication vs. drug preparation** | 0.3656 | ns | <0.000001 | **** | 0.3385 | ns | <0.0001 | **** |
| **equipment vs. drug preparation** | 0.1643 | ns | 0.008001 | ** | 0.0328 | * | <0.0001 | **** |
| **PDMS vs. medication** | <0.0001 | **** | <0.000001 | **** | <0.0001 | **** | 0.0007 | *** |
| **patient vs. medication** | <0.0001 | **** | >0.999999 | ns | <0.0001 | **** | >0.9999 | ns |
| **monitor vs. medication** | >0.9999 | ns | >0.999999 | ns | >0.9999 | ns | 0.0525 | ns |
| **communication vs. medication** | 0.5717 | ns | <0.000001 | **** | >0.9999 | ns | 0.5316 | ns |
| **equipment vs. medication** | 0.2677 | ns | 0.000245 | *** | 0.1939 | ns | 0.0002 | *** |
| **patient vs. PDMS** | >0.9999 | ns | <0.000001 | **** | >0.9999 | ns | 0.1171 | ns |
| **monitor vs. PDMS** | <0.0001 | **** | <0.000001 | **** | <0.0001 | **** | >0.9999 | ns |
| **communication vs. PDMS** | 0.0165 | * | >0.999999 | ns | 0.0034 | ** | >0.9999 | ns |
| **equipment vs. PDMS** | 0.0435 | * | >0.999999 | ns | 0.0525 | ns | >0.9999 | ns |
| **monitor vs. patient** | <0.0001 | **** | >0.999999 | ns | <0.0001 | **** | >0.9999 | ns |
| **communication vs. patient** | 0.0005 | *** | <0.000001 | **** | 0.0002 | *** | >0.9999 | ns |
| **equipment vs. patient** | 0.0015 | ** | 0.00581 | ** | 0.0058 | ** | 0.0575 | ns |
| **communication vs. monitor** | 0.0755 | ns | <0.000001 | **** | 0.7085 | ns | >0.9999 | ns |
| **equipment vs. monitor** | 0.0298 | * | 0.002399 | ** | 0.0825 | ns | >0.9999 | ns |
| **equipment vs. communication** | >0.9999 | ns | 0.458565 | ns | >0.9999 | ns | 0.9996 | ns |

Test results corresponding to Figure 2 analyzed by Friedman’s and Dunn’s multiple comparisons test. P-value < 0.05 considered statistically significant
